# Long Term High Fat Diet Treatment: An Appropriate Approach to Study the Sex-Specificity of the Autonomic and Cardiovascular Responses to Obesity in Mice

**DOI:** 10.3389/fphys.2017.00032

**Published:** 2017-01-26

**Authors:** Thiago Bruder-Nascimento, Obioma J. Ekeledo, Ruchi Anderson, Huy B. Le, Eric J. Belin de Chantemèle

**Affiliations:** Vascular Biology Center, Medical College of Georgia at Augusta UniversityAugusta, GA, USA

**Keywords:** high fat diet, blood pressure, heart rate, vagal tone, vascular adrenergic reactivity, obesity, inflammation, visceral adipose tissue

## Abstract

Obesity-related cardiovascular disease (CVD) involves increased sympathetic activity in men and male animals. Although women exhibit increased visceral fat, metabolic disorders, inflammation and CVD with obesity, whether body weight gain affects autonomic control of cardiovascular function in females remain unknown. Due to the lack of adequate model to mimic the human pathology, this study aimed to develop a murine model, which would allow studying the sex-specificity of the response of the autonomic nervous system to obesity and identifying the origin of potential sex-differences. We tested the hypothesis that sexual dimorphisms in the autonomic response to obesity disappear in mice matched for changes in body weight, metabolic and inflammatory disorders. Male and female C57Bl/6 mice were submitted to control (CD) or high fat diet (HFD) for 24 weeks. Female mice gained more adipose mass and lost more lean mass than males but reached similar visceral adipose mass and body weight, as males, at the end of the diet. 24 weeks of HFD matched male and female mice for visceral adiposity, glycaemia, plasma insulin, lipids, and inflammatory cytokines levels, demonstrating the suitability of the model to study human pathology. HFD did not elevate BP, but similarly increased heart rate (HR) in males (CD: 571 ± 9 vs. HFD: 631 ± 14 bpm, *P* < 0.05) and females (CD: 589 ± 19 vs. HFD: 642 ± 6 bpm, *P* < 0.05). Indices of autonomic control of BP and HR were obtained by measuring BP and HR response to ganglionic blockade, β-adrenergic, and muscarinic receptors antagonists. HFD increased vascular but reduced cardiac sympathetic drive in males (CD: –43 ± 4 and HFD: –60 ± 7% drop in BP, *P* < 0.05). HFD did not alter females' vascular or cardiac sympathetic drive. HFD specifically reduced aortic α-adrenergic constriction in males and lowered HR response to muscarinic receptor antagonism in females. These data suggest that obesity-associated increases in HR could be caused by a reduced cardiac vagal tone in females, while HR increases in males may compensate for the reduced vascular adrenergic contractility to preserve baseline BP. These data suggest that obesity impairs autonomic control of cardiovascular function in males and females, via sex-specific mechanisms and independent of fat distribution, metabolic disorder or inflammation.

## Introduction

The worldwide epidemic of obesity is a leading risk factor for cardiovascular disease (CVD) and a major health burden, in men and women. Due to the protective effects of female sex hormones on the cardiovascular system (Barrett-Connor and Bush, 1991; Mosca et al., 2011), young adult women have been for a long time ignored in clinical cardiovascular study from cohorts and experiments on mechanisms of CVD in basic research (Wizemann and Pardue, 2001; Beery and Zucker, 2011). However, young adult women are severely affected by the current epidemic of obesity (Ogden et al., 2014). They are indeed more prone to obesity than men of the same age and develop more severe forms of obesity (Shields et al., 2011). Notably, the percentage of women suffering from obesity class II and class III is the double of the percentage of men, in the United States and Canada (Flegal et al., 2010; Shields et al., 2011; Ogden et al., 2013, 2014). This rising prevalence of obesity has been associated with a three-fold increase in the incidence of stroke and hypertension and with a stagnation in the number of death from acute myocardial infarction, in women of reproductive age, whereas a decrease is observed in men of the same age (Towfighi et al., 2010, 2011; Wilmot et al., 2015). Despite these alarming data the mechanisms whereby increases in body weight promote CVD in young women remains incompletely understood, and poorly studied.

A shift of the autonomic balance toward an increased sympathetic activity is a common feature of obesity, and a major contributing factor to obesity-related CVD, notably hypertension, in men and male animal models. While it is clearly established that premenopausal women have a lower sympathetic activity than men of the same age (Jones et al., 1996; Matsukawa et al., 1998; Bell et al., 2001; Christou et al., 2005; Messina et al., 2013), it is however unclear whether body weight gain elevates sympathetic activity in females, as it does in males of the same age. Indeed, while a first study reported that muscle sympathetic nerve activity (MSNA) correlates with the percentage of body fat in both young male and female adults (Jones et al., 1996), others did not find correlations between body mass index and MSNA in women (Lambert et al., 2007; Tank et al., 2008). In addition, decreases in body weight have been reported to reduce MSNA in men only (Lambert et al., 2007). Thus, obesity appears to increase MSNA in men, but not or less so in women. The present study aimed to further analyze whether obesity impairs autonomic balance in females, as it does in males and to investigate the mechanism underlying a potential sex difference.

A major complication to the study of the autonomic and cardiovascular responses to diet-induced obesity is the sexual dimorphism in body weight gain, body composition, metabolic alterations, and inflammation. Evidence from the literature indicates that female sex appears to reduce the magnitude of the metabolic and inflammatory disorders induced by chronic HFD treatment (Gupte et al., 2012; Pettersson et al., 2012; Ganz et al., 2014; Singer et al., 2015). This sexual dimorphism in the metabolic and inflammatory responses to short term HFD treatments response may likely contribute to the mild cardiovascular alterations reported in obese female rodents. In opposition to female rodents, young adult women are not protected from obesity-associated metabolic and inflammatory disorders, and appear more prone to obesity-related CVD than men (Thorand et al., 2007; Alemzadeh and Kichler, 2014; Pradhan, 2014; Garcia et al., 2016), which suggests that current murine models of diet-induced obesity are inappropriate and create the need for new animal models better mimicking the human pathology. Therefore, the present study also aimed at developing a mouse model closely mimicking the human scenario, which would allow testing the hypothesis that sex differences in cardiovascular responses to body weight gain disappear in mice matched for changes in body weight, and in metabolic and inflammatory indices. Because obesity in humans is generally a long-term event, this study also aimed to determine if metabolic and inflammatory differences are muted with a prolonged HFD treatment.

## Material and methods

### Animals

All surgical procedures and experimental protocols were approved by the Institutional Animal Care and Use Committee of Augusta University, in accordance with the guide for the care and use of laboratory animals published by National Institute of Health (National Research Council (US) Committee for the Update of the Guide for the Care and Use of Laboratory Animals, 2011). Male and female C57Bl/6 mice (6 weeks of age, Jackson Laboratory, Bar Harbor, ME) were divided in 4 groups and fed either a control diet (CD; BioServ, F6256, Carbohydrate 62%, Fat 17%, Protein 21% kcal, Sodium 0.4%) or a high-fat diet (HFD; BioServ, F2685, 60% of fat calories from lard: Carbohydrate 26%, Fat 59%, Protein 15% kcal, Sodium 0.4%, Diet F3282) *ad libitum*. Tap water was provided *ad libitum*. Based on a pilot study (unpublished data), it was determined that 21 weeks of HFD are required for male and female mice to develop a similar level of obesity. Mice were monitored for 24 weeks. Body weight was measured twice weekly. Food intake was determined in a sub-group of animals placed in metabolic cages for 3 days, 21 weeks after the initiation of the HFD treatment. Mice were housed in an American Association of Laboratory Animal Care–approved animal care facility at Augusta University.

### Body composition

After 21 weeks of control or HFD, body composition was analyzed by nuclear magnetic resonance spectroscopy [EchoMRI (magnetic resonance imaging)](Bruder-Nascimento et al., 2015) and confirmed by direct measurement of fat pad weights (Perigonadal fat pads) at the time of euthanasia (24 weeks).

### *In vivo* blood pressure measurement

After 21 weeks of control or HFD mice were instrumented with telemetry transmitters to record arterial pressure and heart rate (PA-C10, Data Sciences, St Paul, MN). Transmitters were implanted as described previously (Belin de Chantemèle et al., 2009; Huby et al., 2016). After 7 to 14 days of recovery from surgery, necessary for the mice to gain their initial body weight, data were recorded for 7 days. Blood pressure and heart rate values were obtained at 10-min intervals for the duration of the study. Mean values were collected from 24-h averages.

### Indices of autonomic function

Indices of the autonomic function were obtained on the last day of the recording period using a standard pharmacological method involving a single intraperitoneal injection of the ganglionic blocker mecamylamine (5 mg/kg), of the β-adrenergic receptor blocker propranolol (6 mg/kg) and of the muscarinic receptor blocker atropine (1 mg/kg) in conscious mice (Belin de Chantemèle et al., 2009; Huby et al., 2016). Injections were conducted more than 2 h apart in a random order. Maximal blood pressure or heart rate response to blockers within 10 min post-injection was reported. Changes in mean arterial pressure (MAP) or heart rate were expressed as percent of the baseline MAP or heart rate.

### Metabolic profile

Once the blood pressure experiments terminated, fasted mice were anesthetized (isoflurane 5%) and euthanized via decapitation, in accordance with our approved animal protocol. Trunk blood and organs were respectively collected for plasma isolation and weight measurement. Fasting blood glucose and HbA1c levels were measured using a glucometer (AlphaTRAK, Abbott, USA) and A1CNow (Metrika, Terrytown, NY) respectively. Plasma total cholesterol and triglycerides were measured with enzymatic colorimetric assays (Wako, Richmond, VA, USA). Plasma insulin and leptin levels were determined using ELISA kits from ALPCO Diagnostics (Salem, NH, USA) (Belin de Chantemèle et al., 2012).

### Inflammatory profile

Plasma IL-6 and TNF-α levels were measured with EZMIL6 and EZMTNFA ELISA assay kits (Millipore, USA), respectively.

### Vascular reactivity

Thoracic aortas were dissected surgically, cleaned of fat, cut in 2 mm-long rings and mounted on a wire myograph (DMT, Aarhus, Denmark) with 1 g of basal tension (Belin de Chantemèle et al., 2009, 2011). Briefly, 2 tungsten wires were inserted into the lumen of the arteries and fixed to a force transducer and a micrometer. Arteries were bathed in a physiological salt solution and arterial viability was determined with a potassium-rich solution (KCl, 40 mmol/L). Cumulative concentration-response curves to phenylephrine (Phe); (0.1ηmol/L to 100 μmol/L), serotonin (5-HT); (0.1 ηmol/L to 100 μmol/L), acetylcholine (ACh); (0.1 ηmol/L to 100 μmol/L) and sodium nitroprusside (SNP); (0.1 ηmol/L to 10 μmol/L) were conducted. The relaxation in response to ACh and SNP is expressed as percentage of the preconstriction with serotonin (5-HT, 0.1 μM). The response to phenylephrine is expressed as a percentage of KCl-mediated constriction (Belin de Chantemèle et al., 2009, 2011).

### Statistical analysis

All data are expressed as means ± SEM. Data were analyzed using a two-way analysis of variance (ANOVA) followed by a Bonferroni *post-hoc* test comparing sex, diet, and their interaction (GraphPad Prism 7; GraphPad Software Inc., La Jolla, CA). For all comparisons, *P* < 0.05 was considered statistically significant.

## Results

### Metabolic profile

**Figure 1:**
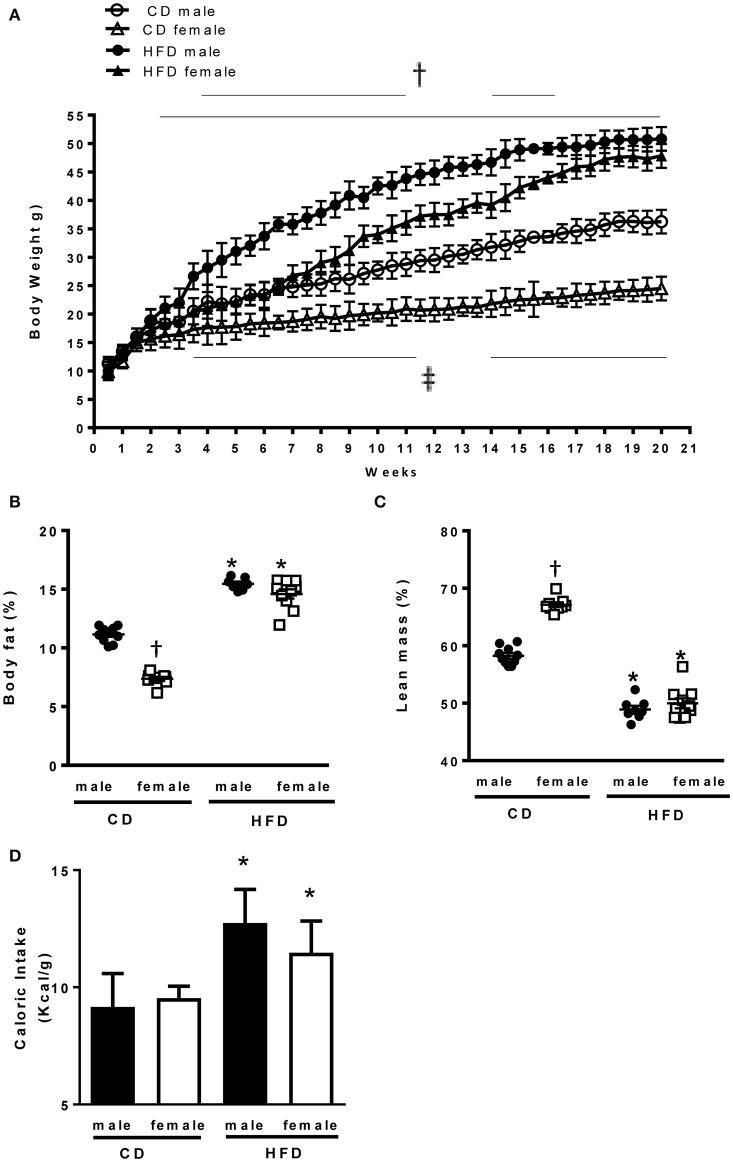
**Effects of 24 weeks of high-fat diet on body weight and body composition in male and female mice. (A)** Body weight of control (CD) and high fat (HFD)-fed mice (g), **(B)** Percentage of body fat and **(C)** lean mass measured by nuclear magnetic resonance spectroscopy (EchoMRI). **(D)** Caloric intake (Kcal x g) in control and high fat-fed male and female mice. Data are presented as mean ± SEM. ^*^P < 0.05 vs. control diet within sex group; ^†^*P* < 0.05 vs. high-fat diet within sex group; ^‡^*P* < 0.05 vs. control male mice. (*n* per group: CD male: 10; CD female: 9; HFD male: 10; HFD female: 10).

At 6 weeks of age male and female mice had comparable body weight (Table 1). In response to CD males presented a larger increase in body weight as compared to females. In response to HFD males exhibited a faster increase in body weight as compared to females, but both male and female mice reached a similar body weight after 21 weeks of HFD treatment (Figure 1A). Body composition analysis via NMR spectroscopy revealed that females had a larger increase in body fat (Figure 1B) and a more pronounced reduction in lean mass (Figure 1C) in response to HFD, as compared to males, despite a similar increase in caloric intake (Figure 1D). Male and females mice exhibited a similar increase in kidney weight with HFD. However, males have a larger increase in heart weight compared to females (Table 1).

**Table 1:** **Body and organs weight**.

	**Male**		**Female**	
	**CD**	**HFD**	**CD**	**HFD**
Initial body weight (g)	11.3 ± 1.2	10.0 ± 1.6	9.9 ± 1.3	10.5 ± 1.5
Final body weight (g)	36.3 ± 4.8	50.8 ± 8.1^*^	24.5 ± 2.9^†^	47.9 ± 7.9^*^
Perigonadal fat/tibia (mg/mm)	73.7 ± 14.3	140.0 ± 17.6^*^	70.5 ± 7.1	150.7 ± 17.1^*^
Heart/tibia (mg/mm)	7.6 ± 0.3	9.2 ± 0.3^*^	7.1 ± 0.5	8.3 ± 0.3^*^
Adrenal/tibia (mg/mm)	0.18 ± 0.02	0.27 ± 0.02^*^	0.22 ± 0.1	0.37 ± 0.1^*^
Kidney/tibia (mg/mm)	18.6 ± 1.2	22.3 ± 0.7^*^	17.7 ± 3.1	21.3 ± 0.9^*^

*Data are expressed as means ± SEM*.

* P < 0.05 vs. respective lean mice;

† *P < 0.05 vs. lean male mice. (n per group: CD male: 7; CD female: 5; HFD male: 5; HFD female: 6)*.

Circulating levels of the adipocyte-derived hormone leptin were similarly increased in male and female mice on the HFD (Table 2). Glycemia, HbA1C, insulin, and cholesterol levels were also similarly increased with HFD in male and female mice (Table 2).

**Table 2:** **Basic physiological and metabolic parameters of control and high-fat diet mice**.

	**Male**		**Female**	
	**CD**	**HFD**	**CD**	**HFD**
Leptin (ng/mL)	4.1 ± 0.9	10.2 ± 0.9^*^	4.7 ± 1.1	11.9 ± 1.4^*^
Insulin (ng/mL)	66.3 ± 17.1	512.6 ± 43.7^*^	59.4 ± 6.9	496.7 ± 54.5^*^
Cholesterol (mmol/L)	0.29 ± 0.04	0.37 ± 0.03^*^	0.27 ± 0.06	0.38 ± 0.04^*^
Triglycerides (mmol/L)	0.89 ± 0.20	0.91 ± 0.19	0.86 ± 0.38	0.87 ± 0.24
Glycemia (mg/dL)	137.0 ± 18.0	196.9 ± 13.0^*^	115.3 ± 21.5	179.10 ± 18.2^*^
Hb1AC	4.7 ± 0.1	5.6 ± 0.3^*^	4.5 ± 0.2	5.8 ± 0.3^*^
IL-6 (pg/mL)	14.2 ± 1.8	40.9 ± 10.0^*^	9.2 ± 3.8	48.2 ± 17.9^*^
TNF-α (pg/mL)	15.1 ± 1.8	47.7 ± 8.4^*^	18.3 ± 1.6	53.2 ± 4.9^*^

*Data are expressed as means ± SEM*.

* *P < 0.05 vs. respective lean mice. (n per group: CD male: 7; CD female: 5; HFD male: 5; HFD female: 6)*.

### Inflammatory profile

As inflammation is a common feature of obesity we measured plasma levels of two cytokines associated with the progression of CVD, IL-6, and TNF-α (Ridker et al., 2000; Gao et al., 2007; Empana et al., 2010; Ammirati et al., 2012), in our 4 groups of mice. These experiments revealed that HFD induced a comparable increase in pro-inflammatory cytokines in male and females mice (Table 2).

### Blood pressure and heart rate

**Figure 2:**
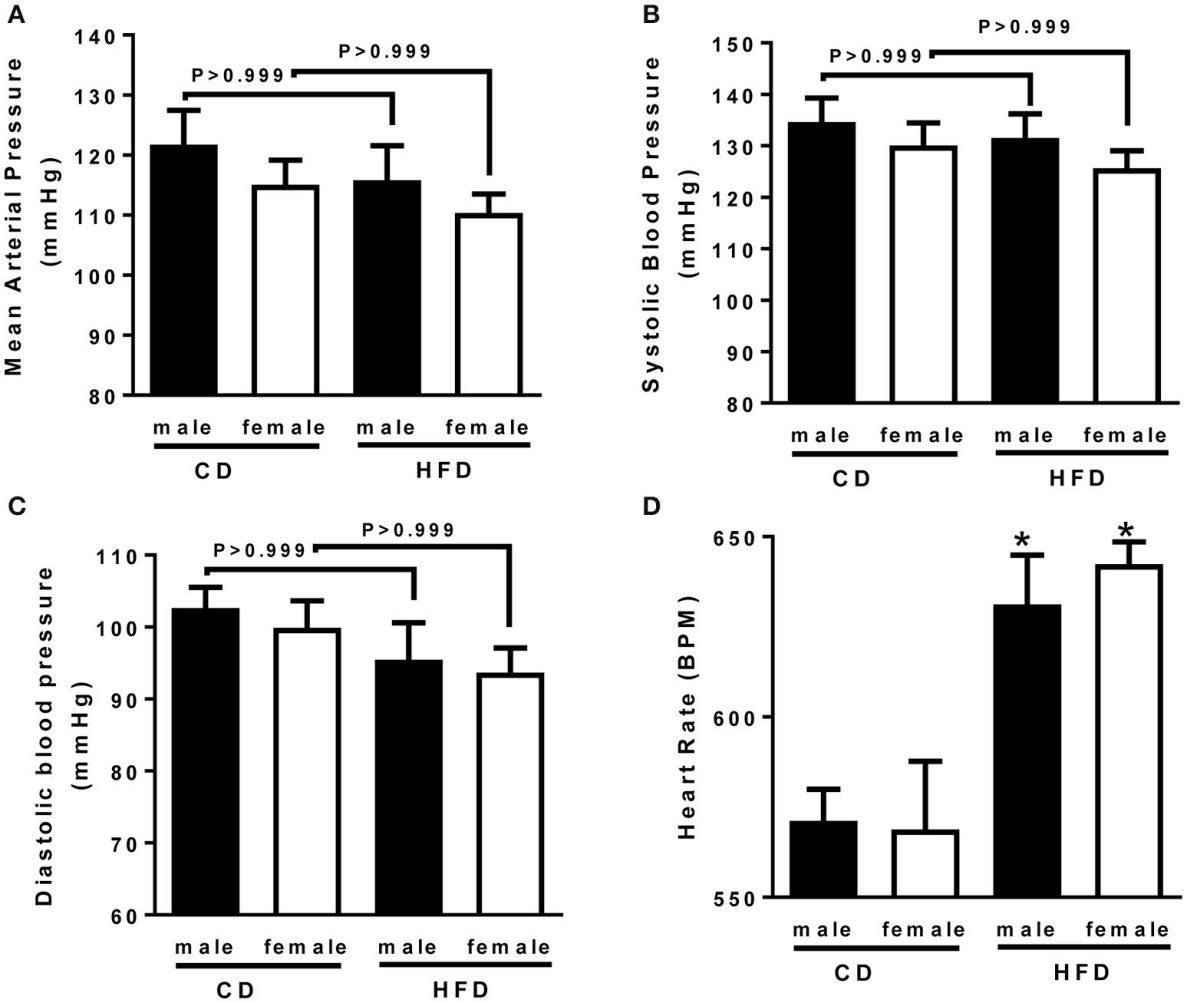
**Twenty-four weeks of high-fat diet preserved blood pressure but increased hear rate in male and female mice. (A)** Mean, **(B)** systolic and **(C)** diastolic blood pressure and, **(D)** heart rate of control (CD) and high-fat diet (HFD) male and female mice, measured by radio-telemetry. Data are presented as mean ± SEM. ^*^P < 0.05 vs. control diet within sex group. (*n* per group: CD male: 6; CD female: 5; HFD male: 6; HFD female: 5)

**Figure 3:**
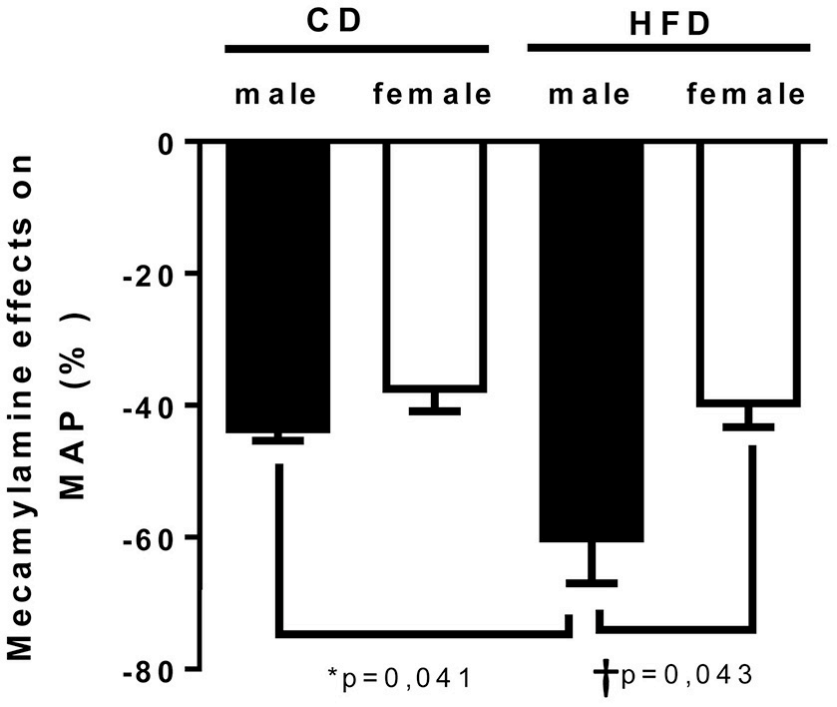
**Twenty-four weeks of high-fat diet increased vascular sympathetic tone in male mice**. Changes in mean arterial pressure in response to mecamylamine [ganglionic blocker (5 mg/Kg i.p)] in control- (CD) and high fat-fed (HFD) male and female mice, measured by radio-telemetry in conscious animals. Data are expressed as percentage of basal condition. Data are presented as mean ± SEM. ^*^P < 0.05 vs. control diet within sex group; ^†^*P* < 0.05 vs. male/ female within diet group. (*n* per group: CD male: 5; CD female: 5; HFD male: 6; HFD female: 5)

**Figure 4:**
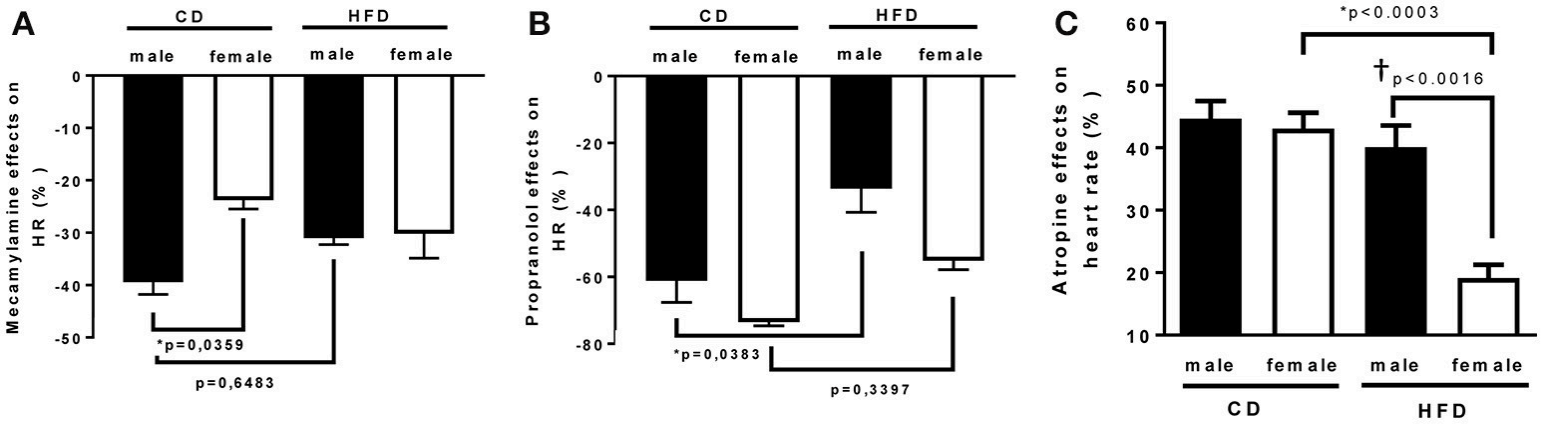
**Twenty-four weeks of high-fat diet decreased cardiac sympathetic tone in male and reduced cardiac vagal tone in female mice**. Changes in heart rate in response to **(A)** mecamylamine [ganglionic blocker (5 mg/Kg i.p)], **(B)** propranolol [β-adrenergic receptor blocker (6 mg/kg)], and **(C)** atropine [muscarinic antagonist (5 mg/Kg i.p)] in control- (CD) and high fat-fed (HFD) male and female mice, measured by radio-telemetry in conscious animals. Data are expressed as percentage of basal condition. Data are presented as mean ± SEM. ^*^P < 0.05 vs. control diet within sex group; ^†^*P* < 0.05 vs. male/ female within diet group. (*n* per group: CD male: 5; CD female: 5; HFD male: 6; HFD female: 5)

To determine whether HFD-induced obesity affects blood pressure, mice were instrumented with radiotelemeter. As reported in Figure 2, no difference in blood pressure was observed between male and female mice on CD. Similarly, HFD did not elevate mean (Figure 2A), systolic (Figure 2B) or diastolic (Figure 2C) blood pressure in either male or female mice. HFD did however significantly raised heart rate to a similar extent in male and female mice (Figure 2D), suggesting a potential impairment in the autonomic control of the cardiovascular function. Autonomic function was assessed acutely, in conscious mice, with randomized injections of sympatholytic drugs and vagal blockers. Blood pressure response to ganglionic blockade revealed a significant increase in vascular sympathetic drive in males submitted to HFD, while obesity was without effect on BP response to ganglionic blockade in females (Figure 3). Ganglionic blockade tended to reduce (Figure 4A), while β-adrenergic receptor antagonism significantly lowered heart rate in male HFD mice (Figure 4B), supporting a reduction in cardiac sympathetic tone with obesity in males. HFD did not alter female's heart rate response to either ganglionic blockade (Figure 4A) or propranolol injection (Figure 4B), suggesting no effects of obesity on cardiac sympathetic tone in female mice. Heart rate response to muscarinic receptor antagonism was used as an index of vagal tone. As reported in Figure 4C, lean male and female mice exhibit similar level of vagal tone. Obesity markedly reduced vagal tone in female mice only (Figure 4C).

### Vascular function

**Figure 5:**
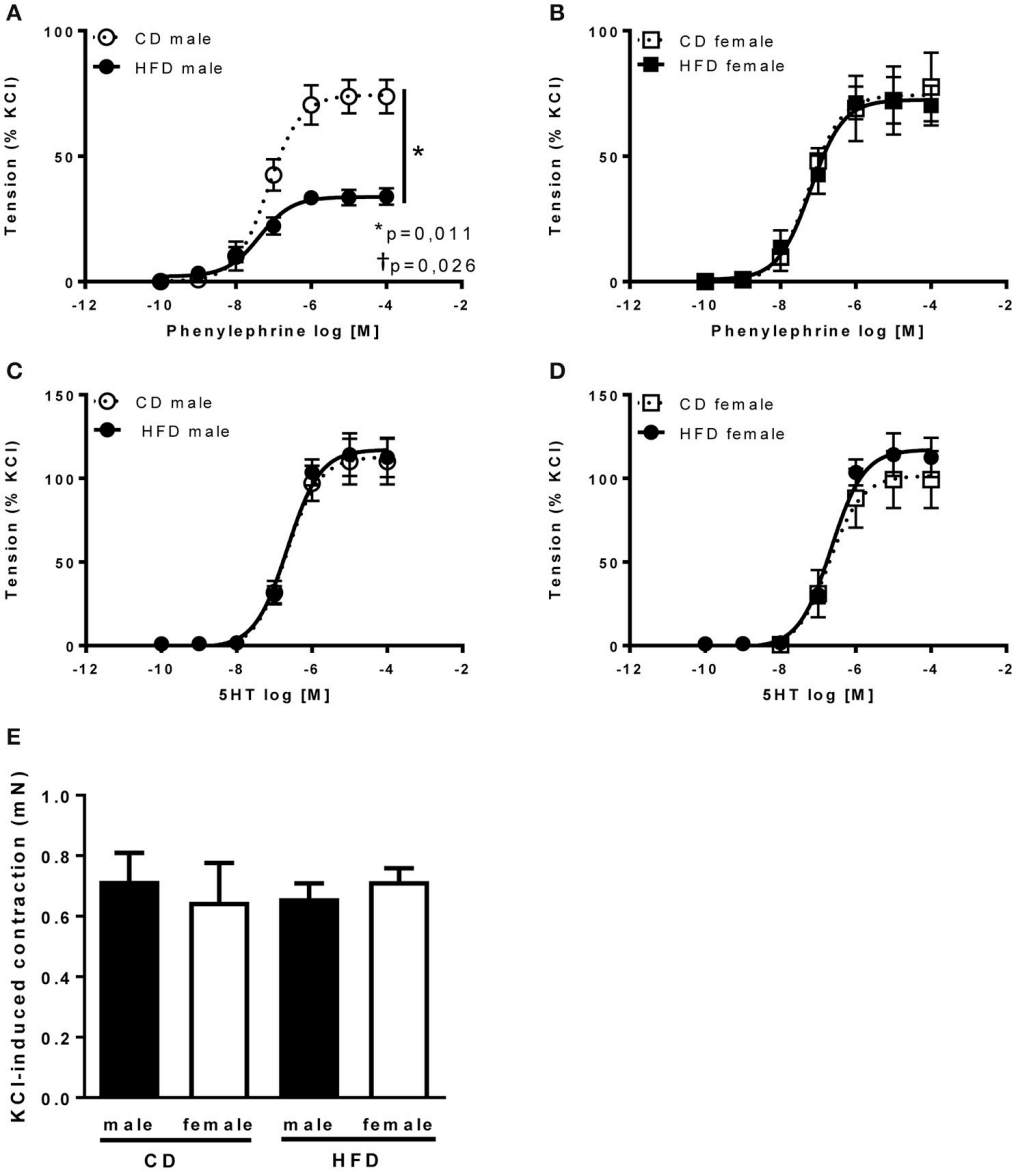
**Twenty-four weeks of high-fat diet reduced vascular adrenergic tone in male mice only**. Cumulative concentration-response curves to phenylephrine and serotonin (5-HT) performed in aortic rings taken from control- (CD) and high fat fed (HFD) male **(A,C)** and female **(B,D)** mice and **(E)** KCl-induced vascular contractility measured in aortic rings taken from control- (CD) and high fat-fed (HFD) male and female mice. Data are expressed as percentage of KCl-induced contractility. Data are presented as mean ± SEM. ^*^P < 0.05 vs. control diet within sex group; ^†^*P* < 0.05 vs. high-fat diet within sex group. (*n* per group: CD male: 5; CD female: 5; HFD male: 5; HFD female: 6).

**Figure 6:**
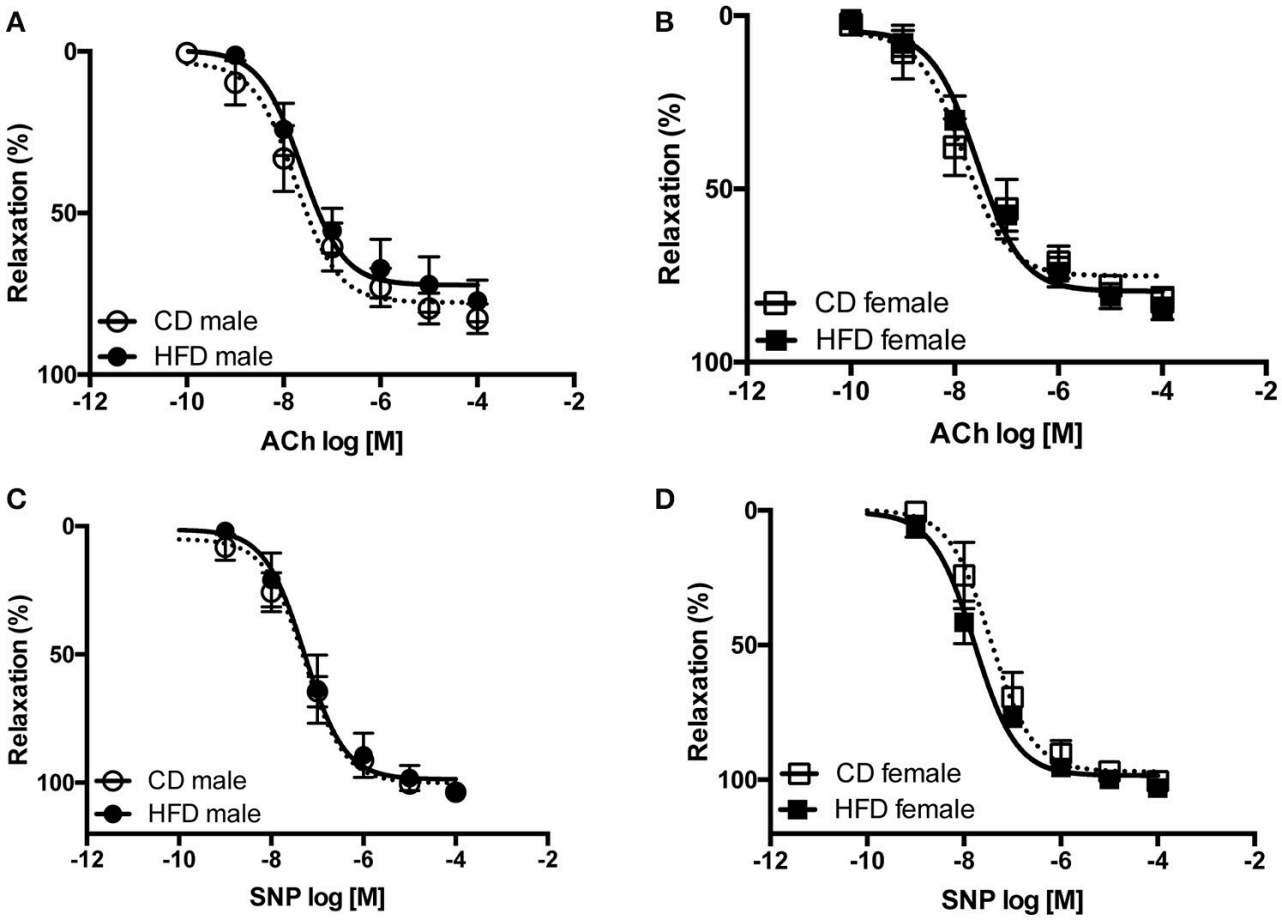
**Twenty-four weeks of high-fat diet preserved vascular relaxation in male and female mice**. Cumulative concentration-response curves to acetylcholine (ACh) and Sodium Nitroprusside (SNP) performed in aortic rings taken from control- (CD) and high fat-fed (HFD) male **(A,C)** and female **(B,D)** mice. Data are presented as mean ± SEM. (*n* per group: CD male: 5; CD female: 5; HFD male: 5; HFD female: 6)

Several studies from our group demonstrate an interaction between sympathetic activity and vascular adrenergic tone, notably report that increased vascular sympathetic tone leads to a reduction in vascular adrenergic contractility (Belin de Chantemèle et al., 2009, 2011; Bruder-Nascimento et al., 2015). Vascular reactivity experiments revealed no difference in vascular adrenergic reactivity between lean male and female mice but reported that HFD markedly reduced aortic constriction to phenylephrine in male mice only (Figures 5A,B). Vascular constriction to serotonin (5-HT) and depolarization (KCl) were neither affected by sex nor the diet (Figures 5C–E). Analysis of the endothelial function via concentration-response curves to acetylcholine (ACh) revealed no difference between males and females and no effects of the diet (Figures 6A,B). Similarly, sex and diet had no effect on endothelium-independent relaxation (SNP, Figures 6C,D).

## Discussion

The present study aimed to characterize the sex-specificity of the response of the autonomic nervous system to obesity and to identify the origin of potential sex differences. We investigate the potential contribution of body weight, metabolic disorders, and inflammation to the autonomic imbalance associated with obesity and tested the hypothesis that the sexual dimorphism in the autonomic response to obesity disappears in mice matched for changes in body weight, metabolic and inflammatory disorders. The main findings of this study are that prolonged HFD treatment: (1) similarly increased body weight in male and female mice and abolished the sexual dimorphism in metabolic and inflammatory responses to obesity observed with short-term HFD treatments, (2) did not elevate blood pressure but increased heart rate to a similar extent in male and female mice, and (3) revealed a sex-specific response of the autonomic nervous system to obesity reflected by an enhanced vascular neurogenic support of blood pressure in males, a lower cardiac sympathetic tone in males and a reduced cardiac vagal tone in female mice. Relevant to these observations is the mouse model of obesity, the dimorphic response of the autonomic nervous system to obesity and the demonstration that, in agreement with our initial hypothesis, similar levels of adiposity, metabolic and inflammatory disorders impair autonomic control of the cardiovascular system in male and female mice.

Compelling evidence from the literature indicates that induction of obesity with a relative short HFD treatment in rodents induces a sexual dimorphism in body weight, fat distribution, metabolic alterations, and degree of inflammation. Indeed, after 8–16 weeks of HFD, female mice are generally leaner and do habitually exhibit reduced increases in body weight, preserved metabolic function and lower degree of inflammation as compared to their male counterparts (Gupte et al., 2012; Pettersson et al., 2012; Ganz et al., 2014; Singer et al., 2015; Jeffery et al., 2016). Amount and distribution of body fat appear as two major factors contributing to the degree of metabolic, inflammatory and cardiovascular disorders associated with obesity (Alvarez et al., 2002; Després, 2012). Therefore, the limited increase in fat mass, mostly restricted to the subcutaneous area (Jeffery et al., 2016), likely explains the absence or the mild cardiovascular disorders developed by female mice submitted to short-term HFD treatments. In opposition to obese female mice, obese women exhibit increased visceral adipose tissue and severe metabolic and inflammatory alterations (Go et al., 2013; Ogden et al., 2013). Thus, the discrepancy between the murine and the human phenotype raises the question of the appropriateness of the murine models currently used. Here we showed for the first time that a long-term HFD treatment abolishes the dimorphic response to HFD revealed with short HFD treatments and provides an appropriate model to study the human scenario. Indeed, female mice initially leaner than males exhibited, with 24 weeks of HFD, larger increases in adipose mass to reached similar levels of visceral fat mass and similar degrees of metabolic dysfunction and inflammation as males, as observed in obese humans. These data indicate that we have generated an adequate murine model and a powerful tool to study the human pathology and investigate whether obesity induces CVD and autonomic imbalance via sex-specific mechanisms.

With this mouse model, no increase in blood pressure with obesity was reported in any of the sexes, despite the long duration of the HFD treatment and the large increases in body weight. Although these data could appear controversial, they are in accordance with previous results by our group and others reporting no elevation in blood pressure in male and female mice submitted to long-term HFD treatments (Belin de Chantemèle et al., 2011; Pettersson et al., 2012; Böhm et al., 2013; Calligaris et al., 2013; Jia et al., 2015; Xu et al., 2015). Despite the lack of increase in blood pressure, we identified sex-specific alterations of the autonomic control of cardiovascular function. We showed that obesity increased vascular but reduced cardiac sympathetic tone in male animals. Obesity was without effects on vascular and cardiac sympathetic tone in female mice but triggered a cardiac parasympathetic withdrawal. Interestingly these sex-specific shifts of the autonomic balance are associated a similar alteration of the cardiac function. Indeed, HFD induced a similar elevation in heart rate in male and female mice. These data are consistent with the human literature reporting increased heart rate in obese patients (Narkiewicz et al., 1999; Nault et al., 2007) and indicating that body weight gain leads to a cardiac parasympathetic withdrawal in a population consisted of a majority of premenopausal women (7 men, 16 women) (Rossi et al., 1989) when weight lost induces a significant increase in cardiac vagal tone, independent of changes in blood pressure, in obese women (Rissanen et al., 2001; Poirier et al., 2003). Thus, reduced cardiac vagal tone appears as a likely explanation for the increased heart rate reported in obese female mice.

HFD did not affect vagal tone in males but, in agreement with the human (Vaz et al., 1997) and rodent literature (Thaung et al., 2015), induced a reduction in cardiac β-adrenergic tone, which has been postulated to be the response to the circulatory overloading (Messerli et al., 1983) brought on by high renal sympathetic nervous activity and sodium retention (Esler et al., 2006; Hall et al., 2015). Therefore, the increased heart rate reported in obese male animals may be the reflection of an increased intrinsic cardiac electrical activity.

In opposition to obese female mice, obese male mice present increased peripheral neurogenic control of blood pressure as shown by a larger decrease in blood pressure in response to acute ganglionic blockade. Consistent with our previous studies (Belin de Chantemèle et al., 2009, 2011; Bruder-Nascimento et al., 2015) we reported that this increase in vascular sympathetic tone is associated with a reduced aortic adrenergic contractility. Although the aorta is a conduit vessel playing minor roles in blood pressure control, it develops, as the resistance arteries, a reduced adrenergic contractility in response to increased sympathetic tone (Belin de Chantemèle et al., 2009, 2011). As suggested previously (Belin de Chantemèle et al., 2011), these data, although indirect, may support a decreased total peripheral resistances in obese male mice. The lack of increase in blood pressure, despite the significant and important elevation in heart rate, represent further arguments to support a decrease in total peripheral resistance in males animals. Indeed, improved renal function and decreased cardiac output appear unlikely in a mouse model that exhibit cardiac and renal hypertrophy, metabolic disorders as well as inflammation. This may indicate that the increase in heart rate reported in obese male animals is likely the combination of compensatory mechanisms developed to preserve physiological blood pressure levels in the presence of reduced total peripheral resistance.

Previous work from our group demonstrated that the reduction in vascular adrenergic reactivity is the direct consequence of an increased sympathetic tone (Belin de Chantemèle et al., 2009, 2011; Bruder-Nascimento et al., 2015). This close interaction between sympathetic tone and vascular adrenergic reactivity explains why obese female mice, that did not exhibit increased vascular sympathetic tone, present a preserved aortic adrenergic contractility. Despite metabolic alterations and inflammation, no endothelial dysfunction was reported in aortic rings from obese male and female mice. A preserved aortic endothelial function in rodents exposed to HFD was reported by our group on diverse occasions (Belin de Chantemèle et al., 2011; Stepp et al., 2013; Huby et al., 2016). It has been postulated that the increased cardiac output associated with obesity (Belin de Chantemèle et al., 2011) and its consequent increase in aortic blood flow and shear stress could contribute to the preserved aortic endothelial function despite the presence of metabolic dysfunctions and increased sympathetic activity (Belin de Chantemèle et al., 2011; Huby et al., 2016).

A major goal of the present study was the address the question of the potential mechanisms leading to the sex-specific response of the cardiovascular system to obesity. We investigated whether adiposity, fat distribution, metabolic disorder, and inflammation could potentially contribute to the development of obesity-related autonomic impairments. We reported that matching male and female mice for body weight, visceral adiposity, glycaemia, plasma lipid profile, and degree of inflammation impaired autonomic control of cardiovascular function in both sexes. However, the nature of the autonomic dysfunction appears sex-specific. Indeed, while males exhibit an increased vascular sympathetic tone, females developed a cardiac parasympathetic withdrawal. The sex-specificity of the autonomic impairment cannot be explained by different metabolic or inflammatory responses to obesity, which suggest the contribution of sex hormones. Estrogen is known to exert sympatho-inhibitory actions (Dart et al., 2002; Hay, 2016) and increase vagal tone (Saleh and Connell, 2000). In addition, long-term HFD treatment has been shown to reduced estrogen levels (Bryzgalova et al., 2008). Therefore, we can speculate that obesity-associated reduction in estrogen levels contributes to the decrease in parasympathetic activity observed in obese female mice. The hypothesis of the estrogen contribution to the sex-specific response of the autonomic nervous system to obesity, would also indicate that, despite reduced, estrogens levels are still high enough to prevent the increase in sympathetic activity associated with increases in visceral adipose tissue (Alvarez et al., 2002). Metabolic hormones such as insulin, the levels of which are significantly increased with obesity, may also contribute to the sex specific response of the autonomic nervous system to obesity, notably play a role in the decreased vagal tone. Indeed, a study conducted in small population of a majority of women (5 men, 11 women) indicated that hypersinsulinemia reduced vagal tone (Van De Borne et al., 1999). Nevertheless, further experiments are required to investigate the respective contribution of sex steroids and metabolic hormones to the autonomic imbalance induced by obesity.

In summary, our data emphasize for the first time the importance of comparing male and female animals of similar weight, fat distribution, and degree of metabolic and inflammatory disorders, in obesity studies, and demonstrate that at least 21 weeks of HFD treatment are required to generate a mouse model that mimics the human pathology. With these very specific conditions, we identified a sex-specific response of the autonomic nervous system to obesity that is neither influence by fat distribution nor by the degree of metabolic and inflammatory disorders but likely requires the intervention of sex steroids. The mouse model generated provides an excellent tool to further study the contribution of sex-steroids to the autonomic response to obesity.

## Author contributions

TB and EB participated in the conception, design of the work, acquisition of the data, analysis and interpretation of the data, redaction of the manuscript. OE, RA, and HL participated in the acquisition, the analysis, and the interpretation of the data.

## Funding

This work was supported by a Scientist Development Grant and an Innovative Research Grant from the American Heart Association (11SDG5060006, 16IRG27770047 to EB), a R01 from the NHLBI (1R01HL130301-01 to EB) and Start-up funds from Augusta University.

### Conflict of interest statement

The authors declare that the research was conducted in the absence of any commercial or financial relationships that could be construed as a potential conflict of interest. The reviewer JC and handling Editor declared their shared affiliation, and the handling Editor states that the process nevertheless met the standards of a fair and objective review.
